# Supplementary material for: Density regulation amplifies environmentally induced population fluctuations
Source: PeerJ. 2023 Feb 2;11:e14701. doi: 10.7717/peerj.14701 (PMC9899430; doi:10.7717/peerj.14701)
Supplement: Supplemental Information 1 [file peerj-11-14701-s001.docx]

Online Supplemental Material

Density regulation amplifies environmentally induced population fluctuations

Crispin M. Mutshinda^1*^, Aditya Mishra^2^, Zoe Finkel^3^, Andrew J. Irwin^1^

*^1^Department of Mathematics and Statistics, Dalhousie University, Halifax NS B3H 4R2, Canada, ^2^Flatiron Institute, New York, NY 10010, USA,* ^3^*Department of Oceanography, Dalhousie University, Halifax NS B3H 4R2, Canada*

*Correspondence:* crispin.mutshinda@dal.ca

This file includes two figures (Figure S1 and Figure S2) as well as a link to the GitHub repository including the R code used to simulate data from the Gompertz model and the OpenBUGS code used to fit the stochastic Gompertz and stochastic Ricker models to simulated data replicates in the simulation study.

Figure S1 displays boxplots of the proportion $\varphi_{dd}=1-\sigma^{2}/v_{\infty}$ of the stationary variance $v_{\infty}$ due to density regulation at different levels at different levels of density regulation ($\beta=0.2, \beta=0.4$ and $\beta=0.6$ ) and different levels of environmental noise (the $\sigma^{2}$value in each panel) in simulated data.


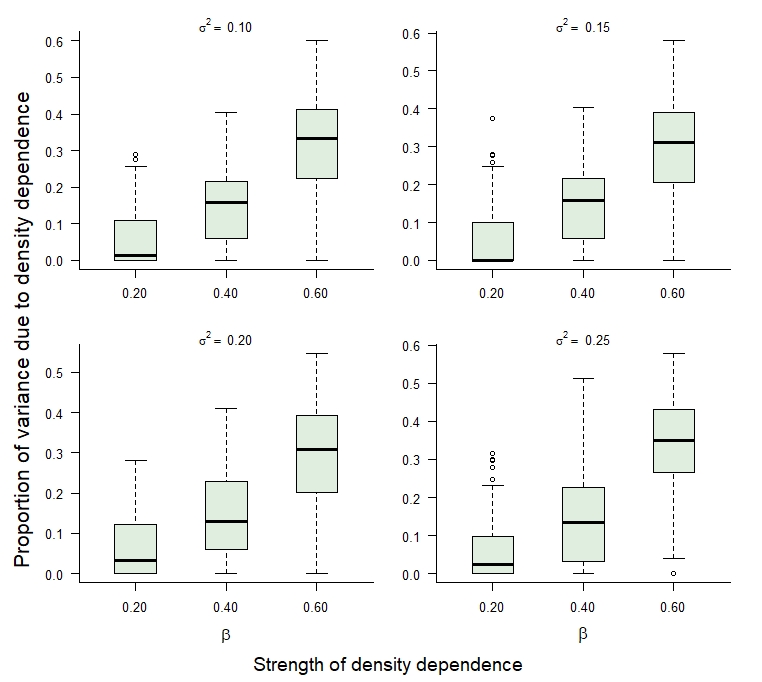


Figure S1 Increasing proportion of stationary variance due density regulation with increasing strength of density dependence.

Box-and-whisker plots summarizing the distributions of the proportion of stationary variance (i.e., the variance of population time series in stationary phase) due to density dependence under the stochastic Gompertz model at different levels of density regulation in simulated population trajectories, showing a monotonic increase in of stationary variance with the strength of density dependence. For each boxplot, the height of the box indicates the 25^th^ ($Q1$) and 75^th^ ($Q3$) per-centiles; the horizontal line inside the box is median, and the lower and upper whisker limits are defined as $Q1-1.5 \times\mathrm{IQR}$ and $Q3 + 1.5 \times\mathrm{IQR}$, respectively, where $\mathrm{IQR}$ represents the interquartile range ($IQR = Q3-Q1$). The dots placed beyond the whiskers’ edges indicate outliers.

In each panel, the proportion of stationary variance attributable to density regulation increased monotonically with the strength of density dependence in simulated population time series


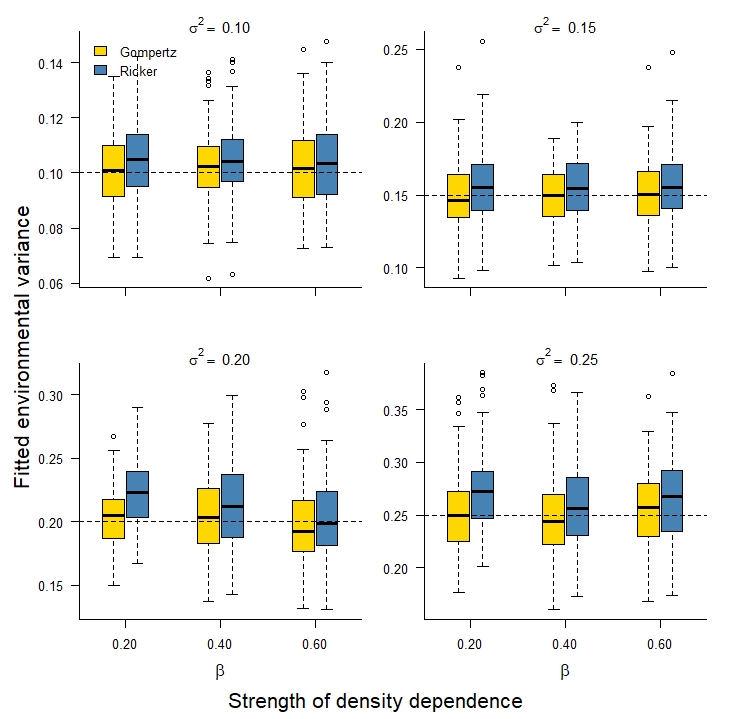


Figure S1: Box-and-whisker plots summarizing the distributions the environmental variance estimated under the stochastic Gompertz model (gold) and stochastic Ricker model (blue) over 100 simulated population trajectories with strengths of density regulation and different levels of environmental noise (the $\sigma^{2}$ value in each panel) in the data. The dashed horizontal line indicates the true value of the environmental noise in simulated data.

The R code for data simulation and the OpenBUGS code used to fit the stochastic Gompertz and stochastic Ricker models to simulated data replicates are available at

https://github.com/mutshinda/Density-Dependence/blob/main/Rcode_DensDep.R
